# Treatment of Exposed Pulps in Filling Teeth

**Published:** 1875-10

**Authors:** Samuel Welchens

**Affiliations:** Lancaster, Pa.


### ARTICLE V.

### *The Treatment of Exposed Pulps in Filling Teeth.*

(Concluded.)

BY SAMUEL WELCHENS, D. D. S., LANCASTER, PA.

Read before the District Dental Society of the Seventh Judicial District of the State of N. Y., at Rochester, N. Y., June 1st and 2d, 1875.

#### THE EXPOSURE OF THE PULP PROPER, AND ITS TREATMENT.

In all dental operations, as in any other branch of surgery, we regard boldness or heroic work and simplicity of treatment as the most successful. The surgeon who cuts nearest the heart with a steady and confiding hand, and is the least ostentatious in his operation, gains the most confidence and establishes the best reputation, because of his simple boldness, which is always a sure guarantee to success. So also in the case of the dental surgeon. He who is most heroic, whose operations are characterized with earnest simplicity, not overburdened with treatment, or jeopardized with complications of his own creation, will, in nine cases out of ten, be successful, whilst the bulk of failures are always on the side of the timid and over-cautious operator, who never gets through healing one disease until another sets in.

The symptoms indicating the approach of caries or an instrument to the pulp need no elaboration in this paper. They are perfectly familiar to an observant, intelligent practitioner and painfully so to the patient. In order, however, fullyt o appreciate all the conditions and changes of our subject, the enumeration of some of them may be of some value here.

The conditions under our first head may not be regarded as strictly pathological. Elongations of the pulp may be approached by caries without any more indications of pain or inconvenience than there is in sensitive dentine. And as if protected by nature, they usually recede as they are approached by such disorder, and only exposed by the sharp cutting edge of the excavatior. In the approach of caries, however, to the pulp proper, where the disease so disintegrates the dentine as to allow thermal changes to effect the pulp, the true diagnosis is by the symptoms. In such cases there is frequent uneasiness, an unpleasant indication of the fact that the tooth is there, slight irritating pain at night, with decided trouble in the application of cold or pressure. These may be regarded as among the most prominent symptoms of the earlier stages of the exposure. When this is the case, a careful excavation of the carious dentine should be made, and if that portion immediately covering the pulp is found to be hard and firm, though dark and decayed, but which affords a pretty secure covering for the pulp, it should be allowed to remain, and after the cavity is carefully and thoroughly cleansed of other particles of carious matter, it should be swabbed with diluted carbolic acid or creosote, after which a thin lining of oxy-chlorids of zinc or Guillois' cement will prepare it for a permanent filling, with very little danger of subsequent trouble.

But we go a step further. When acute inflammation has set in, and the intense suffering of the patient expels every doubt as to the true stage of the disease, and upon a careful examination we find the dentine decomposed and soft, allowing a free application of atmospheric air, and the secretions of the mouth, to change the normal condition of the pulp into a pathological state, the inflammation should be reduced before suppuration sets in by some emollient or antiflogistic application, and when order is again restored, a careful excavation even to an absolute denudation of the pulp should be made, and proper conservative treatment instituted. At this juncture, however, there is some diversity of opinion as to the most effective therapeutical agents in order to restore the pulp to its normal state, and secure its functions to the tooth after the cavity has been perfectly filled.

To reduce the inflammation calendula and aconite, creosote, carbolic acid and glycerine are used respectively, and in mixtures, with more or less success.

My treatment is a preparation of *creosote* and *monarda*, or oil of horse-mint, in equal quantities. This is applied upon a pledget of cotton, and carefully covered with gutta-percha, with two or three re applications in as rapid succession as the case may seem to require, and then a covering or capping is effected with oxy-chloride of zinc or Guillois' cement, and without any delay the case is ready for the filling.

In this treatment, through the rapid recuperative energies of the pulp, we have order and health restored with but slight histological changes. The capping is thus in direct contrast with the substance of the pulp, and the astringent and absorbing powers or properties of the agent employed renders it the best (in our estimation at least,) that can be used. There is no undue pressure, and if the filling is put in as soon as possible, after the cement granulates, and before it becomes saturated with the saliva, and all the conditions of the treatment are intelligently complied with, we have no hesitation in saying that a failure is almost out of the question.

### THE EXTIRPATION OF THE PULP AND TREATMENT OF THE CAVITY.

We now turn to the last stage of exposure and irritation, the treatment of which is much more complicated, and consequently more difficult and protracted.

In "Wedl's Pathology of the Teeth" we have the following description of the disorder to which we now call attention.

"When ultimate denudation of the pulp has taken place, and has continued for some time, the difficulties of the treatment must be very much enhanced ; under these circumstances the surface exposed to the air is much more highly charged with blood than is normally the case, and in many instances is in a constant state of effusing lymph, at times pus—in other words, the surface is an ucler, and should be treated as such; but so precarious is the management of this class of cases that no definite rules can be laid down which will be applicable to all of this description. That which will at one time give relief and permit closure of the cavity, at the next, when the symptoms are similar and the conditions are apparently the same, will be followed by a return of pain, indicating that effused fluids are pressing between the filling and the organ.

"Various irritations have asserted themselves, and probably repeated attacks of pain have been suffered, the consequence of severe congestions. Alterations of structure may have taken place, which will never permit the functions of the pulp to be normally carried on. The important changes which have taken place at the poiut of exposure, where it has been acted upon by various deleterious influences, and along the course of the vessels and nerves, if we are to rely upon the investigation of the German observers in a field which has been surprisingly neglected, are of such a nature as to render questionable a proper cure. The dentinal cells over the exposed part are obliterated. The connective tissue has suffered proliferations The blood vessels are altered by serious structural changes. The nerve-fibres also have been deranged by the depositions of fat granules, not only between them but in their interior. In short all the structural elements of the pulp have undergone changes."

When all this mischief has taken place no one will question the feasibility of devitalizing the pulp and removing it. No man is justifiable in filling over a diseased pulp. If he cannot restore it to a normal state, he should adopt the death penalty at once, and secure the pulp cavity as speedily as possible. Nor is he justfiable in instituting a series of experiments, and take months or a year to do that which can be better done in a few weeks. To submit an individual to the time, torture and expense of a large fancy operation, when the tissue is dead and broken down, or when the pulp is entirely disorganized, without its removal, is not only perpetrating a manifest fraud, but it is flat and consummate quackery.

There are various methods of devitalizing the pulp. But the common nerve paste, composed of arsenic, morphine and creosote, is, I believe, in most common use. In twenty-four hours it will do the work most effectually, when it should be immediately removed and an application of an antiseptic to guard against periostitis. At this juncture the greatest care and judgment should be exercised, lest complications present themselves, or accidents and difficulties arise to retard the work, but in every instance be bold and heroic, be careful and tender, in a word, be master of the situation, and then you will be certain that you are right.

In conclusion, I will submit a few remarks in regard to my method of treating the cavity after I have gotten rid of every vestige of the dead pulp.

If there is an abscess or suppuration of the peridentium, I break down the abnormal tissue with the use of carbolic acdi, pure and simple, and then syringe the cavity with a solution of hypo-sulphite of soda, ten grains to the ounce, and paint the surface of the gum with concentrated tincture of iodine. This treatment for a week will render the case manageable. I then use diluted carbolic acid, about fifteen per cent., or my mixture of creosote and monarda to restore the membrane to a normal status, and fill the root just as soon as this is effected.

I am aware of the fact that much difference of opinion exists as to the method of filling roots after the pulp has been extirpated, and that there is some prejudice against the use of oxy-chloride of zinc for that purpose. I have two methods of filling the pulp cavity in practice. The one with gold, the other with Guillois' cement. I prefer the cement to the oxy-chloride, because it does not set so quickly, thus giving me time to do the work well. My method, in its use, is to cut off a piece of gilling twine about three-quarters of an inch in length, saturate it in a thick paste of the cement, and then, with a fine root plugger, carry it to the apex and pack in; if it does not fill the cavity I add the cement and cover well with cotton until it granulates. If the case has been a plain one, in which there has been no undue inflammatory action, I proceed at once to fill the crown.

If, however, I have had some difficulty in restoring the tissue to a normal condition, I wait a week before permanently closing it up. With this treatment, I scarcely ever fail.

I prefer this to the gold filling for the following reasons:

1. The lint of the twine being non-irritant and saturated with the cement becomes a healthy, solid body, and so medicated to render it incapable of irritation, and by virtue of its absorbing properties, will immediately take up any gas or moisture that may exude from the membrane through the foramen.

2. Gold will not do this; is possessed of no medicament, and if not perfectly solid from the apex to the crown, the exudation will permeate its body, causing disintegration, disease and decay, resulting ultimately in death of the root, abscess, and not unfrequently epulis.—*Missouri Dental Journal.*

---

